# Research Evolution and Frontiers in Glial Scar and Spinal Cord Injury Research: A Bibliometric Perspective

**DOI:** 10.1002/brb3.71681

**Published:** 2026-08-16

**Authors:** Shanyong Zhang, Renfeng Zhang, Yuan Qu, Tingting Hou

**Affiliations:** ^1^ Orthopedics Department The Second Hospital of Jilin University Changchun Jilin China

**Keywords:** Bibliometrics, inflammation, glial scar, neural regeneration, research trends, spinal cord Injury

## Abstract

**Introduction:**

Glial scars play a key role in the pathophysiology of spinal cord injury. This study aims to systematically analyze global research trends and advances on glial scars in spinal cord injury through bibliometric methods.

**Method:**

A comprehensive literature search was performed in the Web of Science Core Collection. Bibliometric analysis using VOSviewer, CiteSpace, and the R package Bibliometrix evaluated publication output, citation patterns, author and institutional contributions, international collaboration, journal impact, keyword co‐occurrence, and emerging research themes.

**Results:**

The analysis encompassed 1293 articles on glial scars and spinal cord injury, authored by 7219 researchers from 4185 institutions across 51 countries. China led with 487 publications (37.7%), followed by the USA with 316 articles and the highest citation count of 27,628. Among institutions, the Chinese Academy of Sciences demonstrated the strongest collaborative network (total link strength = 32). The *Journal of Neuroscience* stood out as the most influential journal, publishing 64 articles and receiving 5703 citations. Keyword co‐occurrence analysis identified five thematic clusters: Injury Responses and Inflammatory Mechanisms; Glial Scar Biology and Central Nervous System Microenvironment; Axonal Regeneration and Inhibitory Molecules; Cell‐Based Therapeutic Strategies; and Proteoglycan‐Related Topics. Burst keyword analysis showed a recent emphasis on terms such as “inflammation,” “recovery,” “delivery,” and “pathway.”

**Conclusion:**

This bibliometric study highlights the substantial growth and diversification of research on glial scars and spinal cord injuries in recent decades. The field is increasingly adopting interdisciplinary approaches and strengthening international collaboration, offering valuable insights into future research directions and potential clinical applications.

## Graphical abstract

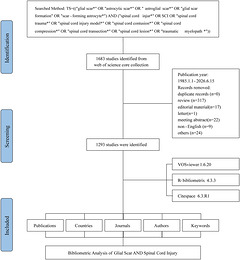

## 1 Introduction

Spinal cord injury remains one of the most devastating neurological conditions, imposing an enormous clinical and socioeconomic burden worldwide. Spinal cord injury often results in lifelong motor, sensory, and autonomic deficits that predominantly affect young individuals, drastically reducing quality of life and productivity (Mensah et al. 2025). Following injury, the central nervous system (CNS) launches a rapid cellular response marked by the activation of glial cells. These cells—primarily astrocytes, along with microglia, oligodendrocyte progenitor cells, and others—undergo phenotypic changes and converge to form a dense glial scar surrounding the lesion (Coutinho Costa et al. 2023). Initially, this scar serves a protective role by containing inflammation and preventing the spread of secondary damage. Over time, however, the glial scar also acts as a formidable barrier to axonal regeneration and functional recovery (Raciti et al. 2025).

The dual role of the glial scar has been the focus of extensive biological research. Reviews and experimental studies have detailed its composition, highlighting the contribution of astrocytes and NG2+ cells and presenting the paradox in which ablation studies that remove specific glial cell populations lead to aggravated tissue degeneration and impaired recovery outcomes (Yang et al. 2020). At the same time, novel therapeutic strategies—including genetic modulation, cell transplantation, and combinatorial approaches—aim to modify scar properties without losing its neuroprotective functions (Pieczonka and Fehlings 2023). Despite significant advances in understanding the molecular mechanisms driving glial scar formation and function, the current literature remains largely qualitative. Many reviews detail signaling pathways (e.g., STAT3 activation), extracellular matrix remodeling, and the role of inhibitory molecules like chondroitin sulfate proteoglycans (CSPGs) (Clifford et al. 2023). However, these studies often lack a comprehensive, quantitative analysis of the evolving research landscape.

Bibliometric methods offer an objective approach that can map out collaboration networks, identify key contributors (countries, institutions, and authors), and reveal emerging trends in scientific investigation (Li et al. 2024). Existing reviews have extensively discussed therapeutic strategies and biological mechanisms related to spinal cord injury and glial scar formation but have primarily focused on qualitative insights (Shafqat et al. 2023; Tran et al. 2018). Notably, no bibliometric analysis has systematically explored the overall research landscape in this area. Therefore, this study aims to conduct a comprehensive bibliometric analysis of publications related to glial scars and spinal cord injury, identifying key contributors, research hotspots, and emerging trends to better inform future investigations and therapeutic strategies in this critical field.

## 2 Materials and Methods

### 2.1 Search Strategies and Data Collection

A comprehensive literature search regarding glial scars and spinal cord injury was performed using the Web of Science Core Collection (WoSCC) (Yeung 2023). The search strategy was formulated as follows: TS = ((“glial scar*” OR “astrocytic scar*” OR “astroglial scar*” OR “glial scar formation*” OR “scar‐forming astrocyte*”) AND (“spinal cord injur*” OR SCI OR “spinal cord trauma*” OR “spinal cord injury model*” OR “spinal cord contusion*” OR “spinal cord compression*” OR “spinal cord transection*” OR “spinal cord lesion*” OR “traumatic myelopath*”)) (Han et al. 2025). The search was restricted to the Science Citation Index Expanded (SCIE) and Social Sciences Citation Index (SSCI) databases. Inclusion criteria were limited to English‐language articles. Literature retrieval was finalized on June 15, 2026, to ensure temporal consistency and minimize discrepancies resulting from database updates, with the publication time frame spanning from 1985, corresponding to the earliest available publication in this field, to June 2026. During data processing, bibliographic records were exported in both “Full Record and Cited References” and “Plain Text” formats. The exported datasets intended for bibliometric analysis encompassed publication and citation metrics, article titles, author affiliations, institutional information, geographic distribution, keywords, and journal sources.

### 2.2 Statistical Analysis

The visualization and analysis of data were conducted utilizing VOSviewer (version 1.6.20), CiteSpace (version 6.3.R1), and the R package Bibliometrix (version 4.4.1). VOSviewer enabled the construction of co‐occurrence and bibliographic coupling networks that included authors, institutions, countries, journals, and keywords, thereby facilitating the identification of research trends and interdisciplinary linkages. The software enabled the visualization of complex academic collaboration networks, elucidating relationships among diverse bibliometric indicators within the research domain.

A comprehensive bibliometric analysis was performed using the R package Bibliometrix. The package facilitated the mapping of global research distribution, the identification of principal trends, and the evaluation of the influence of authors, journals, and countries. The academic impact of researchers and journals was quantified utilizing the h‐index, a robust metric that balances productivity and citation impact (Bertoli‐Barsotti and Lando 2017; Hirsch 2005). Additionally, the g‐index and m‐index were integrated to provide complementary evaluations. The g‐index provides a refined assessment of citation impact by assigning increased weight to highly cited publications (McClelland III et al. 2019). The m‐index normalizes the h‐index relative to the academic career length of the researcher, thus serving as an indicator of scholarly consistency and sustainability (Nocera et al. 2024).

Keyword co‐occurrence analysis was performed using VOSviewer, with “Keywords Plus” selected as the primary data source after comparison with “Author Keywords,” as it provided more comprehensive and accurate results. A minimum occurrence threshold of 10 was applied, and keywords were grouped into clusters based on co‐occurrence patterns. Additionally, CiteSpace was employed for burst keyword detection to identify emerging research trends. The CiteSpace parameters were set as follows: time slicing from January 1985 to June 2026 with one‐year intervals; node type set to keywords; threshold of top 5 per time slice; and pruning via Pathfinder with merged network pruning.

## 3 Results

### 3.1 An Overview of Publications

**FIGURE 1:**
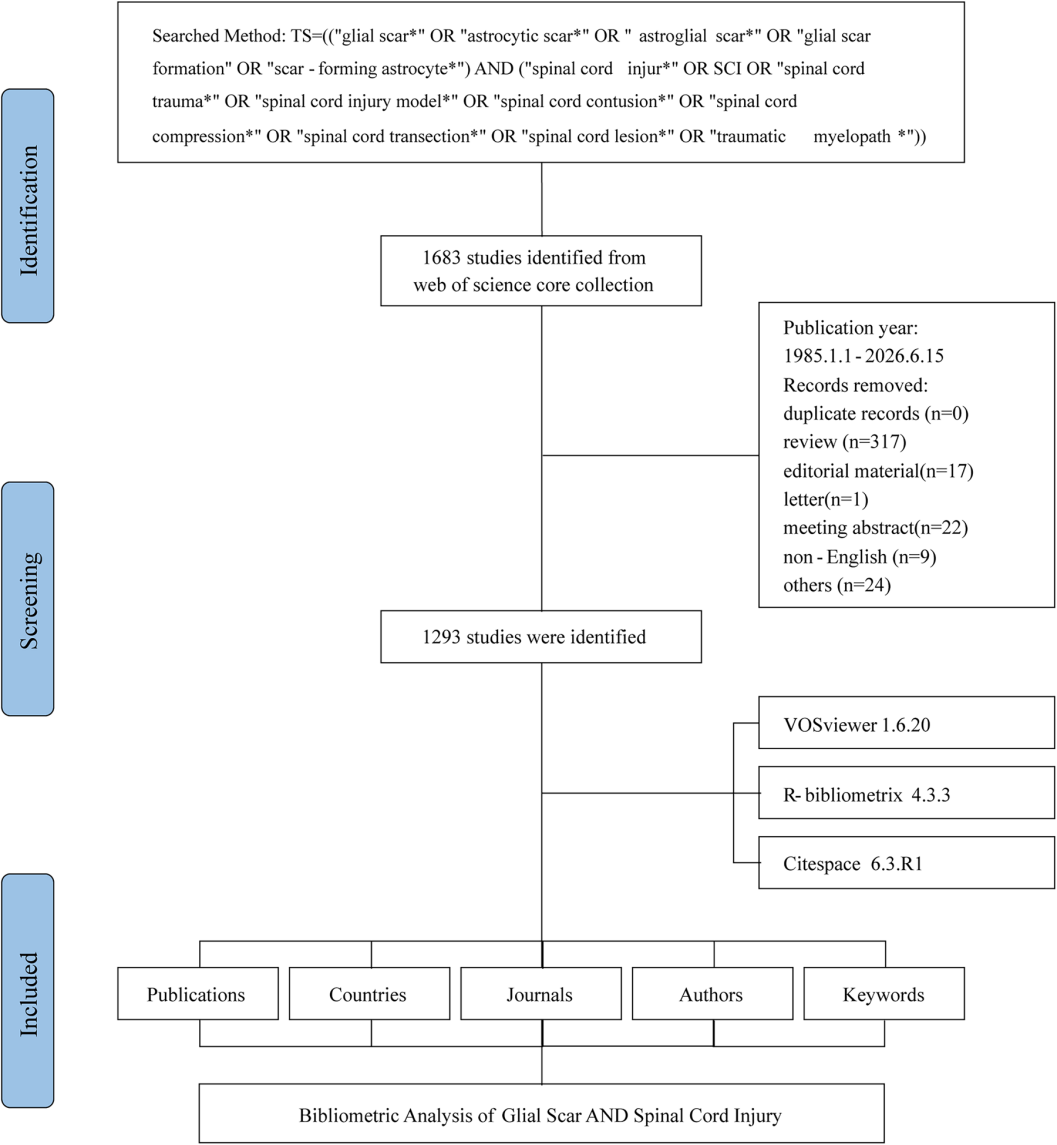
Flowchart of the literature screening process for glial scar and spinal cord injury.

The initial search in the SCIE and SSCI databases yielded 1683 records. After excluding 9 non‐English publications, 1674 records remained. Further exclusion of non‐article document types, including reviews (*n* = 317), editorial materials (*n* = 17), meeting abstracts (*n* = 22), letters (*n* = 1), and other types (*n* = 24), resulted in a final dataset of 1,293 articles for analysis (**Figure** **1**). These works were disseminated across 355 different journals. The contributions originated from 7219 authors affiliated with 4185 institutions spanning 51 countries. Throughout this investigation, the publications collectively referenced 38,631 sources. The average number of co‐authors per article was calculated to be 7.49, with 22.27% of the published works arising from international collaborations. The documents had an average age of 9.85 years, and each article received an average of 53.5 citations. The publications in this domain exhibited an annual growth rate of 9.96%.

**FIGURE 2:**
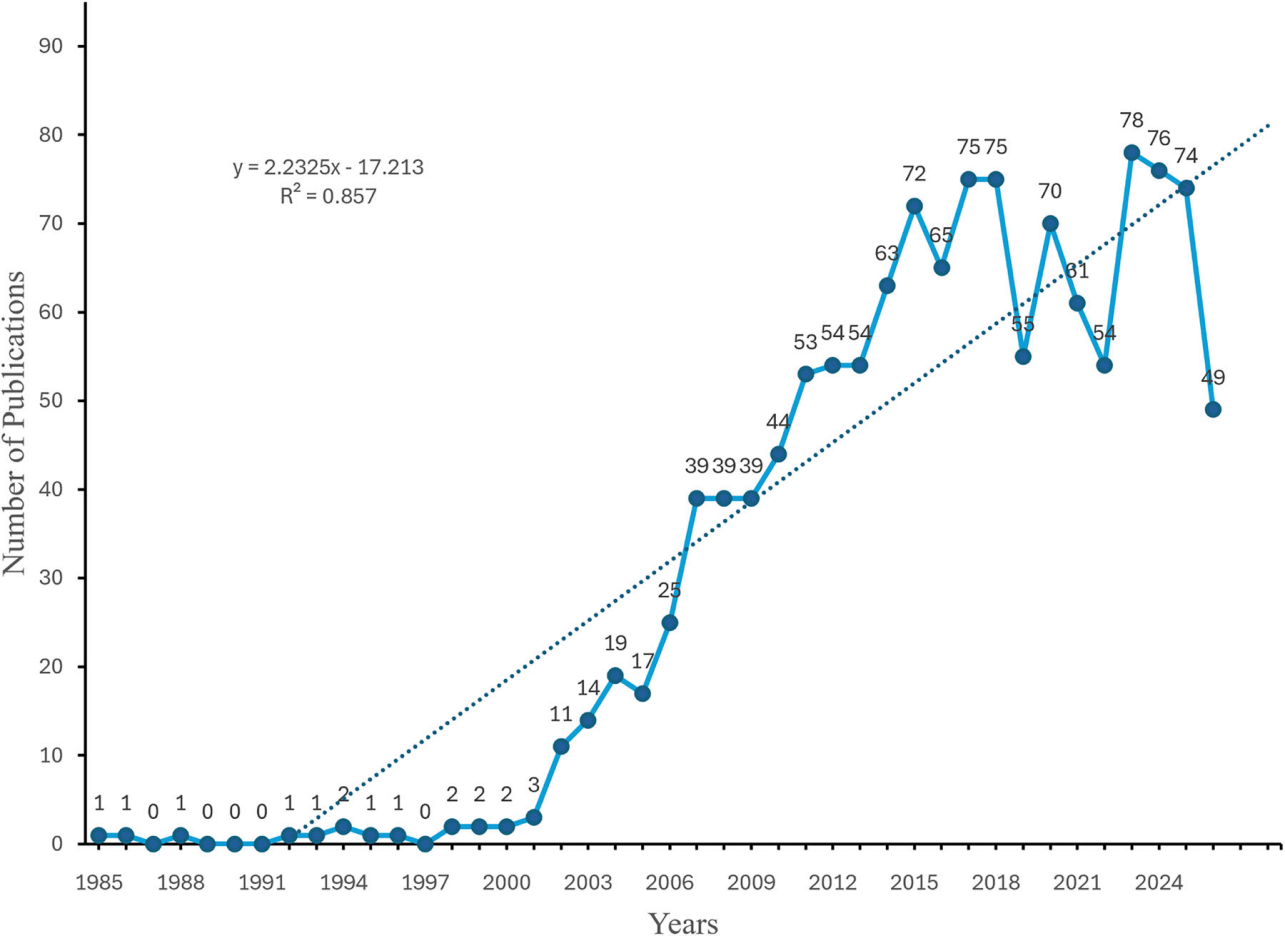
Annual number of publications on glial scar and spinal cord injury (1985–2026).

The annual count of publications revealed a significant upward trajectory over the years (**Figure** **2**). The field began with a single article in 1985 and encountered modest growth throughout the early years. In the early 2000s, a marked increase became evident, characterized by a steady rise in publication numbers. The period from 2011 to 2018 was especially noteworthy, with annual output consistently exceeding 50 articles and reaching peaks of 75 publications in both 2017 and 2018. The highest annual output was observed in 2023, with 78 publications. This upward trend continued with slight fluctuations. The most cited article in this field, titled “Chondroitinase ABC promotes functional recovery after spinal cord injury,” was published in *Nature* (impact factor [IF] = 56.1) in 2002 and accumulated a total of 1955 citations (https://doi.org/10.1038/416636a). The second most cited article, titled “Traumatic spinal cord injury,” was published in *Nature Reviews Disease Primers* (IF = 79.8) in 2017 and accumulated 1875 citations (https://doi.org/10.1038/nrdp.2017.18). The third most cited article, titled “Astrocyte scar formation aids central nervous system axon regeneration,” was published in *Nature* (IF = 56.1) in 2016 and accumulated 1488 citations (https://doi.org/10.1038/nature17623).

### 3.2 Analysis of the Countries

**FIGURE 3:**
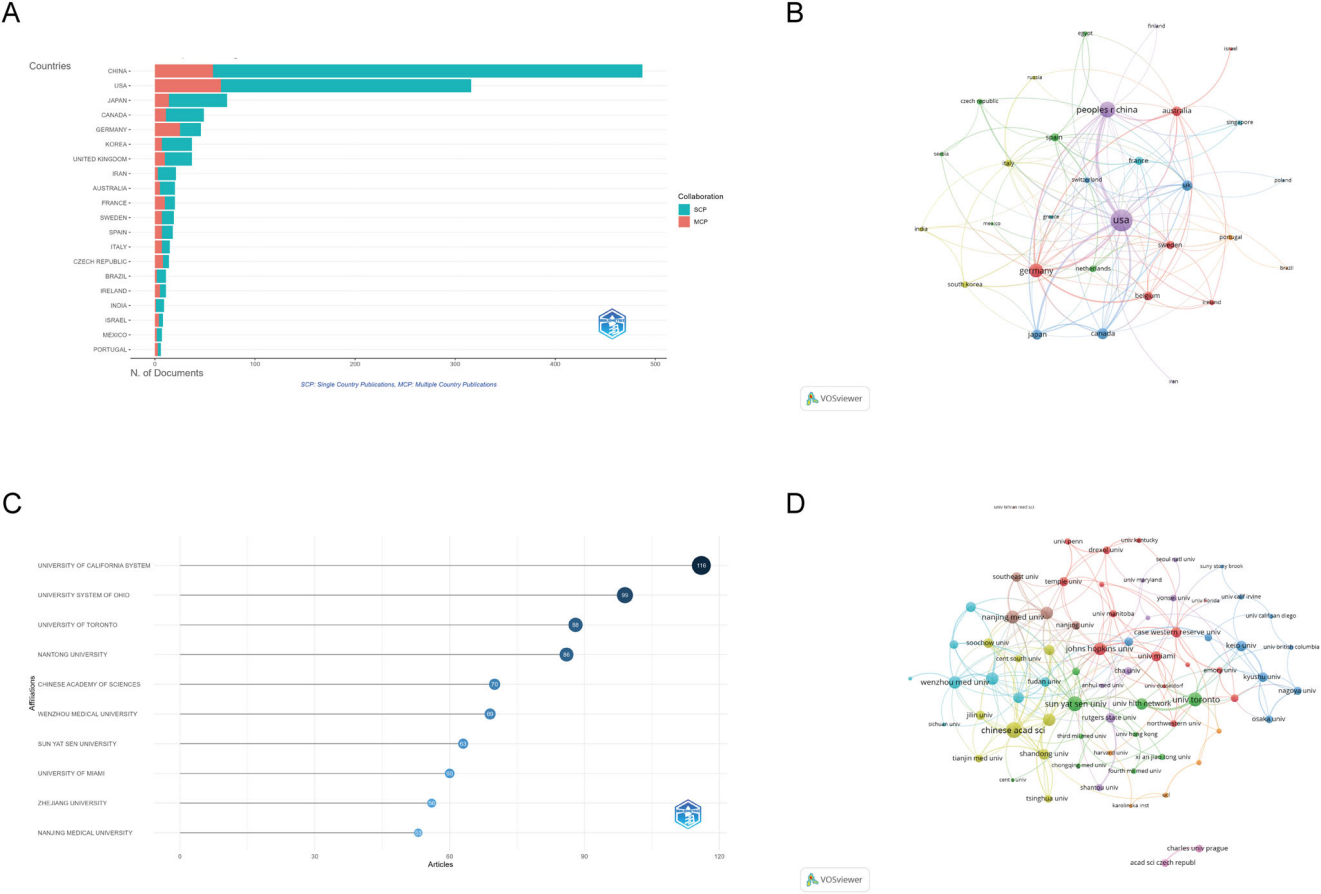
Analysis of countries and institutions. (A) Distribution of corresponding author's publications by country. SCP: single country publications. MCP: multiple country publications. (B) Visualization map depicting the collaboration among different countries. (C) Top ten institutions by article count and rank. Circle size shows article count. Darker shades indicate higher ranks. (D) Visualization map depicting the collaboration among different institutions.

China was at the forefront of research output in glial scar and spinal cord injury (SCI) studies, contributing 487 articles, which accounted for 37.7% of the total publications. The United States of America (USA) followed with 316 articles, ranking second in terms of total publications (TP = 1056) and leading in total citations (TC = 27,628). Japan ranked third, producing 72 articles with a citation count of 4,342. Notably, Canada, with 49 publications, boasted the highest average citations per article at 120.5, surpassing other leading countries (**Figure** **3A** and **Table** **1**).

**TABLE 1:** Publication and citation profiles of leading countries.

Country	Articles	Freq	SCP	MCP	MCP_Ratio	TP	TP_rank	TC	TC_rank	Average Citations
CHINA	487	0.377	429	58	0.119	1742	1	13,911	2	28.6
USA	316	0.244	250	66	0.209	1056	2	27,628	1	87.4
JAPAN	72	0.056	58	14	0.194	266	3	4342	4	60.3
CANADA	49	0.038	38	11	0.224	189	4	5906	3	120.5
GERMANY	46	0.036	21	25	0.543	154	5	3509	6	76.3
KOREA	37	0.029	30	7	0.189	146	6	883	10	23.9
UNITED KINGDOM	37	0.029	27	10	0.270	121	7	3703	5	100.1
IRAN	21	0.016	18	3	0.143	105	8	454	15	21.6
AUSTRALIA	20	0.015	15	5	0.250	82	9	930	9	46.5
FRANCE	20	0.015	10	10	0.500	67	11	951	8	47.5
SWEDEN	19	0.015	12	7	0.368	57	13	1458	7	76.7
SPAIN	18	0.014	11	7	0.389	80	10	550	14	30.6
ITALY	15	0.012	8	7	0.467	57	12	658	11	43.9
CZECH REPUBLIC	14	0.011	6	8	0.571	36	18	605	12	43.2
BRAZIL	11	0.009	9	2	0.182	37	17	280	16	25.5
IRELAND	11	0.009	6	5	0.455	39	16	134	23	12.2
INDIA	9	0.007	8	1	0.111	26	22	221	17	24.6
ISRAEL	8	0.006	4	4	0.500	21	23	568	13	71.0
MEXICO	7	0.005	5	2	0.286	45	14	84	27	12.0
PORTUGAL	6	0.005	3	3	0.500	30	20	184	20	30.7

Articles: publications of corresponding authors only. Freq: frequency of total publications. SCP: single country publications. MCP: multiple country publications. MCP_Ratio: proportion of multiple country publications. TP: total publications. TP_rank: rank of total publications. TC: total citations. TC_rank: rank of total citations. Average Citations: average number of citations per publication.

Among the 30 countries that participated in global partnerships, each contributing a minimum of five articles, the USA exhibited the highest number of collaborations, with a total link strength of 195. China followed with 101 total link strength, while Germany ranked third with a total link strength of 79 (**Figure** **3B**).

### 3.3 Analysis of the Institutions

Among the 72 institutions involved in international collaborations with a minimum of eight articles, the Chinese Academy of Sciences (CAS) had the highest number of partnerships, recording a total link strength of 32. Sun Yat‐sen University followed with a total link strength of 29, and the University of Toronto ranked third with a total link strength of 25 (**Figures** **3C** and **3D**).

### 3.4 Analysis of Journals

The *Journal of Neuroscience* ranked first with an h‐index of 57, the highest number of publications at 64, and total citations reaching 5,703, accompanied by an IF of 4.3 (Journal Citation Reports [JCR] Q2). *Experimental Neurology* ranked second with 63 articles, an h‐index of 33, and 3,499 citations, achieving an IF of 4.8 (Q1). The *Journal of Neurotrauma* ranked third with 48 publications, an h‐index of 26, and total citations of 2,165, with an IF of 3.8 (Q2). *Biomaterials*, although having a lower publication count at 25 articles, ranked highly with an h‐index of 22 and an impressive IF of 13.6 (Q1), while accumulating 1128 citations. Other notable journals included *Glia* and *PLOS ONE*, with h‐indices of 22 and 20 and 27 publications each. *Glia* had 1,497 citations (ranked fifth) and an IF of 5.5 (Q1), while *PLOS ONE* had 720 citations and an IF of 2.8 (Q2) (**Table** **2**).

**TABLE 2:** Bibliometric indicators of high‐impact journals.

Journal	h_index	IF	JCR	g‐index	m‐index	PY_start	TP	TP_rank	TC	TC_rank
*JOURNAL OF NEUROSCIENCE*	57	4.3	Q2	64	1.966	1998	64	1	5703	1
*EXPERIMENTAL NEUROLOGY*	33	4.8	Q1	55	1.000	1994	63	2	3499	2
*JOURNAL OF NEUROTRAUMA*	26	3.8	Q2	42	1.040	2002	48	3	2165	3
*BIOMATERIALS*	22	13.6	Q1	25	1.048	2006	25	9	1128	9
*GLIA*	22	5.5	Q1	27	0.647	1993	27	6	1497	5
*PLOS ONE*	20	2.8	Q2	27	1.111	2009	27	8	720	18
*JOURNAL OF NEUROSCIENCE RESEARCH*	19	3.1	Q3	27	0.543	1992	27	7	1111	11
*NEURAL REGENERATION RESEARCH*	18	8.5	Q1	29	0.947	2008	47	4	457	28
*MOLECULAR NEUROBIOLOGY*	17	5.5	Q1	29	1.133	2012	29	5	322	36
*EUROPEAN JOURNAL OF NEUROSCIENCE*	15	2.5	Q3	19	0.455	1994	19	12	931	13
*JOURNAL OF NEUROINFLAMMATION*	15	11.5	Q1	17	1.000	2012	17	14	508	26
*BRAIN RESEARCH*	14	3.2	Q3	19	0.636	2005	19	11	980	12
*ACTA BIOMATERIALIA*	13	10.4	Q1	13	0.929	2013	13	17	306	39
*NEUROSCIENCE*	13	3.3	Q2	20	0.542	2003	20	10	810	17
*JOURNAL OF COMPARATIVE NEUROLOGY*	12	2.3	Q1	13	0.293	1986	13	19	860	15
*JOURNAL OF NEUROCHEMISTRY*	12	4.6	Q1	14	0.462	2001	14	16	592	21
*BIOCHEMICAL AND BIOPHYSICAL RESEARCH COMMUNICATIONS*	11	2.5	Q3	11	0.478	2004	11	24	243	48
*NEUROSCIENCE LETTERS*	11	2.2	Q3	16	0.355	1996	16	15	496	27
*SCIENTIFIC REPORTS*	11	4.9	Q1	18	0.917	2015	18	13	345	33
*MOLECULAR AND CELLULAR NEUROSCIENCE*	10	3.4	Q2	11	0.400	2002	11	25	578	22

h_index: the h‐index of the journal, which measures both the productivity and citation impact of the publications. IF: impact factor. JCR: Journal Citation Reports quartile ranking (Q1: top 25%, Q2: 25%‐50%, Q3: 50%‐75%, Q4: bottom 25%). g‐index: gives more weight to highly cited articles. m‐index: the h‐index divided by the number of years since the first published paper. TP: total publications. TP_rank: rank of total publications. TC: total citations. TC_rank: rank of total citations. PY_start: publication year start.

**FIGURE 4:**
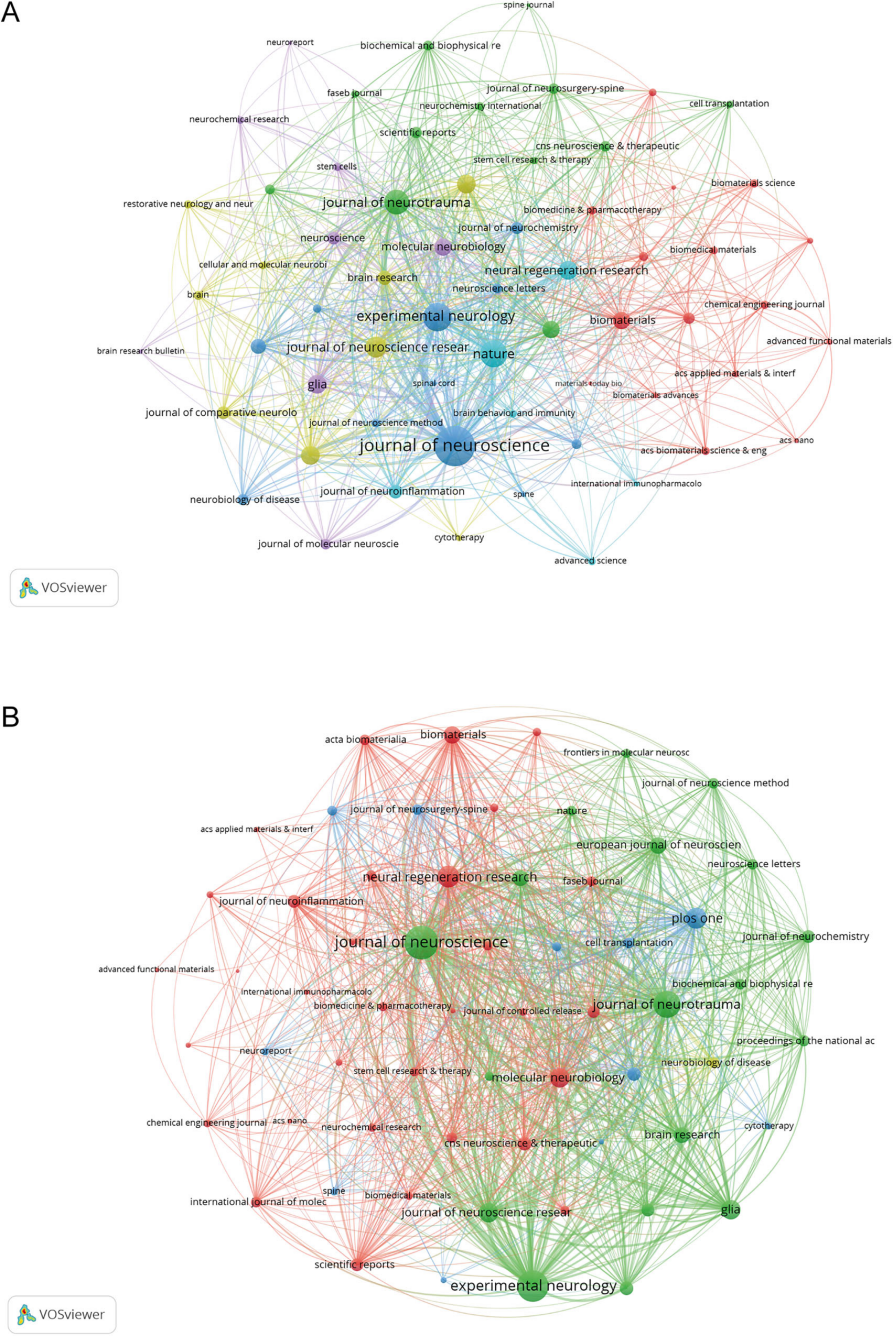
Analysis of journals. (A) Journal co‐occurrence network diagram. (B) Journal bibliographic coupling network diagram.

The co‐occurrence network of journals included **62** journals with a minimum of five occurrences (**Figure** **4A**). Among these, the *Journal of Neuroscience* was identified as having the highest total link strength of 843, followed by *Experimental Neurology* with 415, and *Nature* with 380. In the bibliographic coupling network, which also comprised 62 journals each having at least five coupled links (**Figure** **4B**), the *Journal of Neuroscience* exhibited the strongest total link strength at 54,238, while *Experimental Neurology* and the *Journal of Neurotrauma* ranked second and third, with total link strengths of 46,911 and 33,623, respectively.

### 3.5 Analysis of the Authors

The leading authors in this research domain were ranked according to their h‐index (**Table** **3**). Dai Jianwu was recognized at the top with the highest publication count of 24 (ranked first), an h‐index of 15, and a total of 1307 citations. Fehlings Michael G. followed closely with 15 publications, an h‐index of 14, and the highest citation total of 3535 (ranked second). Silver Jerry ranked third, contributing 16 articles, with an h‐index of 14 and total citations of 2591. Xiao Zhifeng also produced 18 publications, maintaining an h‐index of 13 and achieving 1,026 citations.

**TABLE 3:** Publication and citation profiles of high‐impact authors.

Author	h_index	g‐index	m‐index	PY_start	TP	TP_Frac	TP_rank	TC	TC_rank
DAI JIANWU	15	24	0.88	2010	24	2.22	1	1307	10
FEHLINGS MICHAEL G.	14	15	0.74	2008	15	2.57	5	3535	2
SILVER JERRY	14	16	0.74	2008	16	2.14	3	2591	3
XIAO ZHIFENG	13	18	0.77	2010	18	1.53	2	1026	13
ZHAO YANNAN	12	16	0.71	2010	16	1.33	4	992	14
WANG WEI	11	14	0.52	2006	14	1.71	6	733	18
CHEN BING	10	12	0.59	2010	12	0.95	7	749	16
JENDELOVA PAVLA	10	10	0.63	2011	10	1.28	10	485	29
NAKAMURA MASAYA	10	10	0.48	2006	10	1.09	11	1435	6
OKANO HIDEYUKI	10	10	0.48	2006	10	1.09	12	1435	6
LI XING	9	12	0.82	2016	12	1.19	8	640	21
SCHACHNER MELITTA	9	11	0.45	2007	11	1.99	9	291	43
XIAO JIAN	9	10	0.82	2016	10	0.79	13	482	30
FADEN ALAN I.	8	8	0.40	2007	8	1.74	17	621	24
FISCHER ITZHAK	8	8	0.38	2006	8	1.49	20	524	27
KADOMATSU KENJI	8	8	0.38	2006	8	0.92	21	784	15
KARIMI‐ABDOLREZAEE SOHEILA	8	8	0.47	2010	8	1.83	22	1314	9
KESSLER JOHN A.	8	8	0.42	2008	8	1.07	23	1155	12
KUBINOVA SARKA	8	8	0.62	2014	8	0.86	24	375	36
LIU YANG	8	9	0.50	2011	9	1.15	15	413	33

h_index: the h‐index, which measures both the productivity and citation impact of the publications. g‐index: gives more weight to highly cited articles. m‐index: the h‐index divided by the number of years since the first published paper. TP: total publications. TP_Frac: fractional total publications. TP_rank: rank of total publications. TC: total citations. TC_rank: rank of total citations. PY_start: publication year start.

**FIGURE 5:**
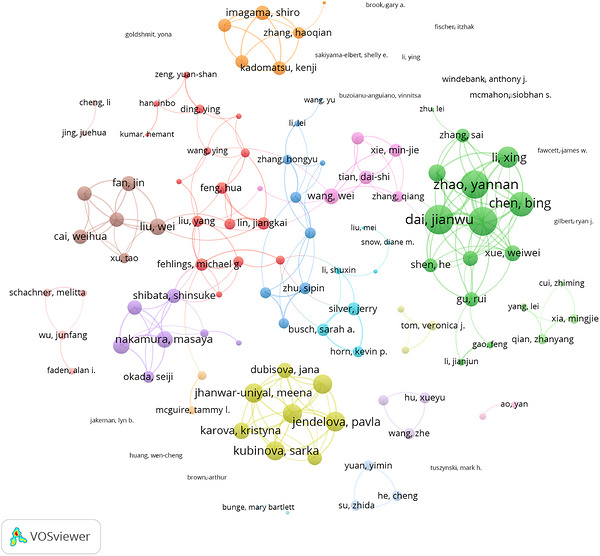
Visualization map depicting the collaboration among different authors.

The collaboration network among authors engaged in international partnerships, each contributing a minimum of five articles, was depicted in **Figure** **5**. Dai Jianwu had the highest number of collaborative connections, with a total link strength of 79, followed by Xiao Zhifeng (75) and Zhao Yannan (73).

### 3.6 Analysis of the Keywords

**FIGURE 6:**
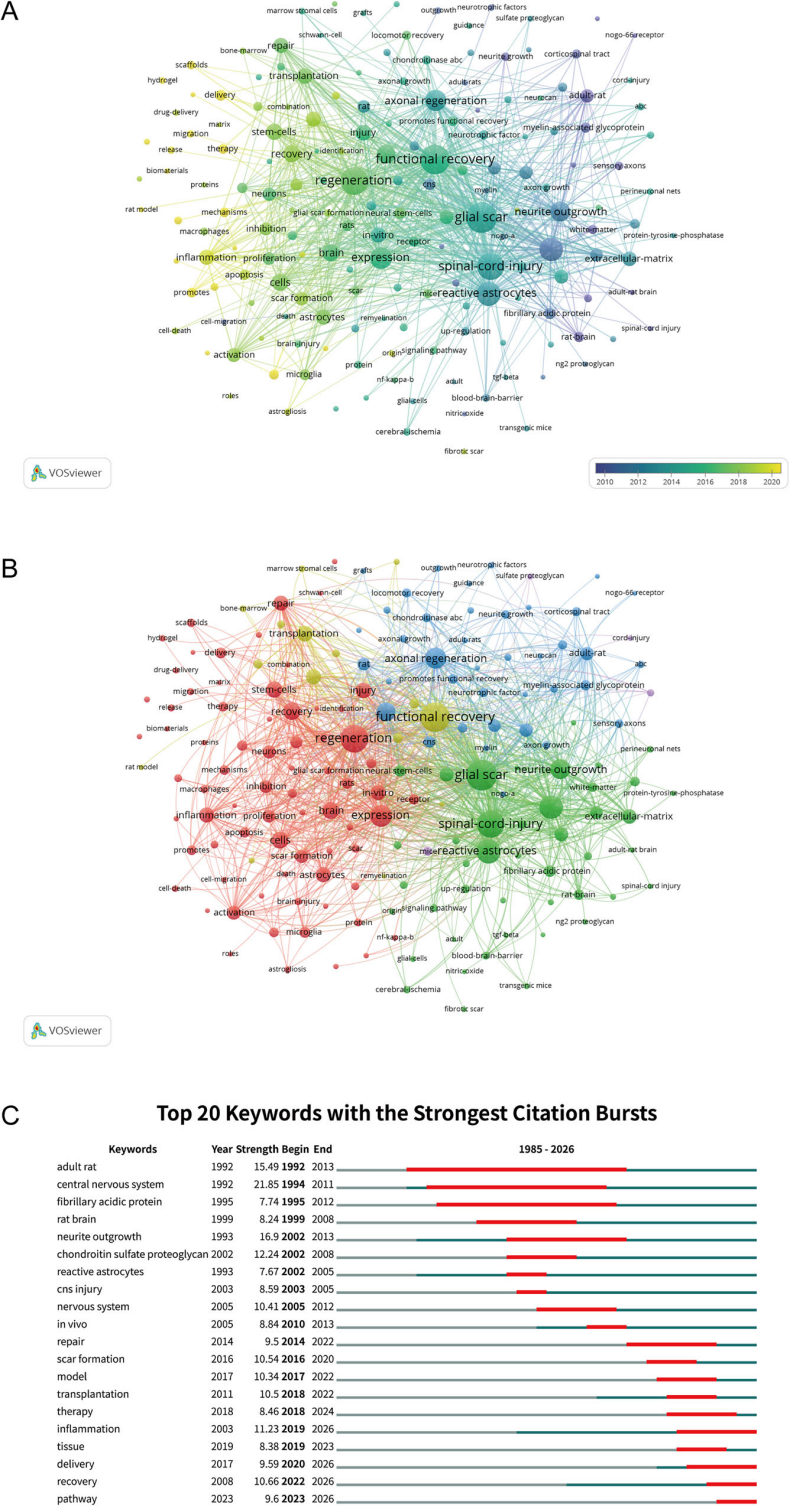
Visual analysis of keywords. (A) Keyword cluster analysis (VOSviewer). (B) Keyword co‐occurrence network analysis with overlay visualization depicting temporal evolution of research themes. (C) Top 20 keywords with the strongest citation bursts (CiteSpace).

In total, 175 keywords with a minimum occurrence of 10 were identified. Keyword clustering analysis using VOSviewer revealed five distinct clusters (Figure 6A). Cluster 1, the largest with 73 items, centered on injury responses and inflammatory mechanisms, featuring terms such as “inflammation,” “apoptosis,” “astrocytes,” “microglia,” and “activation.” Cluster 2 encompassed 44 items related to glial scar biology and the central nervous system (CNS) microenvironment, including “glial scar,” “reactive astrocytes,” “extracellular matrix,” “blood‐brain barrier,” and “fibrotic scar.” Cluster 3 contained 39 items focusing on axonal regeneration and inhibitory molecules, highlighted by terms such as “axonal regeneration,” “chondroitinase ABC,” “corticospinal tract,” and “myelin‐associated glycoprotein.” Cluster 4 comprised 15 items centered on cell‐based therapeutic strategies, including “functional recovery,” “transplantation,” “mesenchymal stem‐cells,” “Schwann‐cells,” and “differentiation.” Cluster 5, the smallest with 4 items, addressed proteoglycan‐related topics, featuring “proteoglycans,” “sulfate proteoglycans,” “cord injury,” and “mice.”

The network visualization of keyword co‐occurrence, overlaid with average publication year, illustrated the temporal evolution of research themes (Figure 6B). Keywords appearing in darker shades (earlier years) such as “neurite outgrowth,” “adult‐rat,” and “central‐nervous‐system” represented foundational research topics. Keywords in lighter shades (more recent years) such as “inflammation,” “therapy,” “delivery,” and “scaffolds” indicated the progressive shift toward translational and therapeutic research.

### 3.7 Analysis of Burst Keywords

The evolution of research trends in glial scar and SCI was reflected by citation bursts of keywords over time (**Figure** **6C**). In the early period (1985‐2013), prominent terms included “adult rat” (strength = 15.49, 1992–2013) and “central nervous system” (21.85, 1994–2011), along with “neurite outgrowth” (16.9, 2002–2013). During the transitional period (2002–2013), “chondroitin sulfate proteoglycan” (12.24, 2002–2008) and “reactive astrocytes” (7.67, 2002–2005) emerged as prominent burst terms. In the more recent period (2014‐2026), keywords such as “model” (10.34, 2017–2022), “delivery” (9.59, 2020–2026), and “therapy” (8.46, 2018–2024) showed strong bursts. Additionally, “inflammation” (11.23, 2019–2026), “recovery” (10.66, 2022–2026), “scar formation” (10.54, 2016–2020), and “pathway” (9.6, 2023–2026) also emerged as burst terms. Notably, “inflammation,” “delivery,” “recovery,” and “pathway” remained active burst keywords through 2026, indicating these as the current research frontiers in this field.

## 4 Discussion

This study conducted a comprehensive bibliometric analysis of the literature surrounding glial scars and spinal cord injury (SCI), revealing significant insights into research trends and thematic evolution in this critical field. A total of 1293 articles published between 1985 and June 2026 were analyzed, encompassing contributions from 7219 authors across 4,185 institutions in 51 countries. The keyword co‐occurrence analysis identified five distinct clusters, highlighting the multifaceted nature of current research, including injury responses and inflammatory mechanisms, glial scar biology and the central nervous system (CNS) microenvironment, axonal regeneration and inhibitory molecules, cell‐based therapeutic strategies, and proteoglycan‐related topics. This thematic mapping indicates a shift from traditional neuroanatomical studies to a more nuanced understanding of biological responses and therapeutic interventions, particularly as terms related to “repair,” “delivery,” and “transplantation” have emerged prominently in recent years.

Publication output exhibited steady growth over the study period, with annual output first exceeding 10 articles in 2002 and consistently surpassing 50 articles per year from 2011 onward. The annual growth rate of 9.96% reflects sustained interest in this research domain. Notably, the mean citations per article peaked for publications from 1998 (343.0 citations per article) and 2002 (290.2 citations per article), coinciding with landmark discoveries in chondroitin sulfate proteoglycan (CSPG) degradation and axonal regeneration strategies that have shaped the trajectory of subsequent research.

China dominated global publication volume with 487 articles (37.7%), reflecting substantial investment in neuroscience research and extensive academic collaborations (Xie et al. 2014). However, the USA led in total citations (27,628) and average citations per article (87.4), suggesting higher per‐article impact. Canada, despite contributing only 49 articles, achieved the highest average citations (120.5), indicating a pattern of producing highly influential studies. The relatively low multiple country publication (MCP) ratio of China (11.9%) compared with Germany (54.3%), the Czech Republic (57.1%), and France (50.0%) suggests that strengthening international collaborative networks could further enhance the global impact of Chinese research in this field (De Lima et al. 2021). Among institutions, the Chinese Academy of Sciences (CAS) demonstrated the strongest collaborative network (total link strength = 32), followed by Sun Yat‐sen University (29) and the University of Toronto (25), underscoring the role of these institutions as central hubs facilitating knowledge exchange across research groups. Dai Jianwu emerged as the most prolific author with 24 publications and an h‐index of 15. He developed 3D‐bioprinted neural tissue constructs using a novel bioink, showing potential for spinal cord repair (Liu et al. 2021). Fehlings Michael G., despite a lower publication count (15 articles), accumulated the second highest total citations (3535), reflecting his foundational contributions to translational SCI research (Hejrati and Fehlings 2021).

The *Journal of Neuroscience* was identified as the most influential publication venue, leading in both total publications (64) and total citations (5703), with the highest co‐occurrence link strength (843) and bibliographic coupling strength (54,238). This dual dominance in both co‐occurrence and coupling networks indicates that the *Journal of Neuroscience* serves as a central knowledge hub connecting fundamental neuroscience and translational SCI research. *Experimental Neurology* and the *Journal of Neurotrauma* also featured prominently, while high‐impact materials science journals such as *Biomaterials* (IF = 13.6) and *Acta Biomaterialia* (IF = 10.4) reflected the growing intersection between biomaterial engineering and neural regeneration strategies.

### 4.1 Hotspots

The keyword co‐occurrence analysis revealed five distinct thematic clusters, each representing critical dimensions of glial scar and SCI research.

### 4.2 Cluster 1: Injury Responses and Inflammatory Mechanisms

This largest cluster (73 items) encompasses the cellular and molecular events triggered by SCI, including astrocyte activation, microglial responses, apoptosis, and inflammatory cascading. Evidence suggests that insufficient stimulation of axon/neurite growth may contribute more to regeneration failure than the glial scar itself (Shafqat et al. 2023; Tran et al. 2018). NF‐kappa‐B signaling regulates astrocytic inflammatory gene expression and interacts with Janus kinase/signal transducer and activator of transcription (JAK/STAT) pathways to influence repair outcomes (Ageeva et al. 2024). In a contusion SCI rat model, neutralizing IL‐20 reduced astrocytic scarring and inflammatory signaling, improving locomotor recovery on the BBB scale (Lee et al. 2020). The prominence of terms such as “inflammation,” “apoptosis,” “microglia,” and “oxidative stress” within this cluster reflects the recognition that modulating the inflammatory microenvironment is essential for achieving meaningful functional recovery. Burst keyword analysis further confirmed this trend, with “inflammation” maintaining a strong citation burst (strength = 11.23) that persists through 2026.

### 4.3 Cluster 2: Glial Scar Biology and CNS Microenvironment

This cluster (44 items) focuses on the cellular composition and molecular architecture of the glial scar and its surrounding microenvironment. Experimental studies describe a phenotypic transition from naive to reactive to scar‐forming astrocytes, maintaining a chronic scar environment that constrains regeneration and functional recovery (Tamaru et al. 2023). Astrocyte‐specific Yes‐associated protein (YAP) is activated after SCI; conditional knockout reduces astrocyte proliferation and scar formation but also impairs axonal regeneration and behavioral recovery (Xie et al. 2020). The inclusion of terms such as “blood‐brain‐barrier,” “fibrotic scar,” and “extracellular‐matrix” highlights the complexity of the post‐injury CNS microenvironment, where multiple cell types and matrix components interact to determine regenerative outcomes. Scar‐forming astrocytes exhibit a dual role by limiting lesion spread while inhibiting axonal regrowth, as observed in SCI and ischemic stroke (Yang et al. 2020). Understanding the transition between protective and inhibitory phases of glial scar formation remains a central challenge, as reflected by the sustained burst of “scar formation” (strength = 10.54, 2016–2020).

### 4.4 Cluster 3: Axonal Regeneration and Inhibitory Molecules

This cluster (39 items) addresses the molecular barriers to axonal regrowth and strategies to overcome them. Multiple studies show that degrading CSPGs with chondroitinase ABC enhances axon growth and functional recovery after SCI by mitigating glial scar‐associated inhibition (Hu et al. 2021; Bradbury et al. 2002; Hunanyan et al. 2009). However, the challenge lies in translating these findings from preclinical models to clinical settings. While CSPG degradation has been shown to improve axonal regeneration in laboratory settings, the inconsistent results in clinical trials suggest that merely targeting the extracellular matrix may not be sufficient (Muir et al. 2019). The presence of myelin‐associated inhibitory molecules, including Nogo‐A, myelin‐associated glycoprotein (MAG), and their receptors (Nogo‐66 receptor, protein‐tyrosine‐phosphatase sigma [PTP‐sigma]) within this cluster indicates that the inhibitory environment following SCI is multifactorial. Recent studies have demonstrated that combinatorial approaches targeting both CSPGs and myelin‐associated inhibitors yield superior regenerative outcomes compared with single‐target strategies (Serradj and Hollis 2025). The keyword “corticospinal tract” further underscores the emphasis on functionally relevant long‐tract regeneration as a key outcome measure.

### 4.5 Cluster 4: Cell‐Based Therapeutic Strategies

This cluster comprising 15 items reflects increasing emphasis on translational strategies. Mesenchymal stem cells and their extracellular vesicles can suppress astrocyte activation, reduce glial fibrillary acidic protein (GFAP) and CSPG expression, and inhibit glial scarring through anti‐inflammatory effects (Xia et al. 2023). In chronic SCI models, surgical modification of the GFAP‐positive scar combined with NT‐3‐chitosan implantation promoted reconnection of neural pathways and functional recovery (Zhao et al. 2022). Transplanted human astrocytes derived from glial‐restricted progenitors can support regeneration by modifying the inhibitory microenvironment and promoting axonal growth (Haas and Fischer 2013). The co‐occurrence of “functional recovery” with “transplantation,” “schwann‐cells,” and “differentiation” within this cluster indicates that cell‐based therapies are increasingly evaluated not only for their capacity to replace lost neural tissue but also for their ability to modulate the glial scar microenvironment. The burst keyword “transplantation” (strength = 10.5, 2018–2022) highlights a period of intensified investigation into cell‐based interventions, while the sustained burst of “delivery” (strength = 9.59, 2020–2026) suggests growing attention to optimizing the methods by which therapeutic agents and cells are delivered to the injury site (Zhang and Shi 2026).

### 4.6 Cluster 5: Proteoglycan‐Related Topics

This smallest cluster (4 items) centers on proteoglycans and their role in the inhibitory post‐injury environment. The spinal injury scar contains multiple cell types and extracellular matrix molecules, including CSPGs such as NG2, which contribute to neuron entrapment and limit regeneration. Targeting CSPG production and modulation offers new therapeutic avenues for overcoming the inhibitory effects of the glial scar (Bradbury and Burnside 2019). Although compact, this cluster represents a foundational biochemical theme that intersects with multiple other clusters, particularly regarding the molecular mechanisms underlying axonal growth inhibition and the development of enzyme‐based therapeutic strategies.

### 4.7 Research Frontiers and Emerging Trends

Keyword burst analysis indicates shifting priorities over time. The overlay visualization of keyword co‐occurrence networks demonstrated a clear temporal transition from foundational topics (darker shades representing earlier average publication years) toward translational and therapeutic themes (lighter shades representing more recent years). In recent years (2014–2026), terms such as “model,” “delivery,” and “therapy” have become prominent. Four burst keywords remained active through 2026: “inflammation” (strength = 11.23), “recovery” (10.66), “delivery” (9.59), and “pathway” (9.6), collectively delineating the current research frontiers. Research increasingly addresses inhibitory components of glial scars, including CSPGs and inflammatory factors, as well as mechanisms related to oxidative stress (Ashammakhi et al. 2019) and associated pathways (Clifford et al. 2023). A modified scaffold promotes neurogenesis by directing the differentiation of injury‐activated endogenous neural stem cells at the injury site, demonstrating the potential of engineered biomaterials to stimulate neurogenesis in the glial scar environment (Li et al. 2017). The convergence of biomaterial engineering, immunomodulation, and cell‐based therapies suggests that future breakthroughs will likely emerge from combinatorial strategies that simultaneously address multiple aspects of the post‐injury inhibitory environment (Sousa et al. 2025). Bhatt et al. reported how blood‐spinal cord barrier disruption initiates cytokine‐driven astrogliosis that culminates in scar formation and axonal growth inhibition, emphasizing temporal dynamics linking innate immune signaling to extracellular matrix deposition that consolidates the mature scar (2024). These findings underscore the need for temporally coordinated therapeutic interventions that account for the evolving nature of the post‐injury microenvironment.

### 4.8 Limitations

This study had several limitations that should be acknowledged. First, the reliance on the WoSCC may introduce selection bias, as it primarily includes publications indexed in this database, potentially overlooking relevant articles in other databases such as PubMed, Scopus, and Embase. Second, the focus on English‐language publications might exclude valuable insights from research conducted in non‐English‐speaking regions, thereby limiting the representativeness of the findings. Third, citation‐based metrics may be influenced by self‐citation and other citation behaviors, which could affect the evaluation of research impact. Fourth, recently published articles may not have accumulated sufficient citations to reflect their true impact, potentially underestimating contributions from the most recent years. Finally, the bibliometric approach is inherently descriptive and cannot substitute for systematic reviews or meta‐analyses in evaluating the quality of evidence or the efficacy of specific therapeutic interventions.

## 5 Conclusion

This bibliometric analysis delineates the dynamic landscape of research on glial scar and SCI, identifying five distinct thematic clusters: injury responses and inflammatory mechanisms, glial scar biology and CNS microenvironment, axonal regeneration and inhibitory molecules, cell‐based therapeutic strategies, and proteoglycan‐related topics. Recent citation bursts underline an increasing focus on innovative therapeutic strategies, including the roles of inflammation, delivery systems, and newly emerging concepts like oxidative stress and signaling pathways. The sustained activity of burst keywords “inflammation,” “recovery,” “delivery,” and “pathway” through 2026 identifies these as the most promising research frontiers. These findings serve to guide future research initiatives toward developing integrated treatment approaches that combine immunomodulatory, biomaterial‐based, and cell‐based strategies to address the multifaceted inhibitory environment following SCI.

## Author Contributions

Renfeng Zhang: Writing – original draft, Writing – review and editing, data curation, formal analysis. Tingting Hou: conceptualization, Writing – original draft, Writing – review and editing. Yuan Qu: formal analysis, Writing – original draft, Writing – review and editing, data curation. Shanyong Zhang: Writing – original draft, Writing – review and editing, project administration.

## Funding

This study was supported by the Science and Technology Research Project of the Education Department of Jilin Province (JJKH20241333KJ). The funders played no role in the design, conduct, or reporting of this study.

## Conflicts of Interest

The authors declare no conflicts of interests.

## Data Availability

All data generated or analyzed during this study are included in this article.
